# Cutaneous reactions to COVID‐19 vaccine at the dermatology primary care

**DOI:** 10.1002/iid3.568

**Published:** 2021-11-27

**Authors:** Martina Burlando, Astrid Herzum, Claudia Micalizzi, Emanuele Cozzani, Aurora Parodi

**Affiliations:** ^1^ Department of Health Sciences (DISSAL), Section of Dermatology, San Martino Polyclinic Hospital IRCCS, Largo R University of Genova Genova Italy

**Keywords:** coronavirus vaccine, COVID‐19 skin, COVID‐19 urticaria, COVID‐19 vaccination reaction, COVID‐19 vaccine rash, vaccine allergy

## Abstract

**Introduction:**

Coronavirus disease 2019 (COVID‐19) vaccines can cause adverse reactions, mainly from vaccine‐induced immune responses. Some of these may also involve the skin and worry unaware patients. A better understanding of such adverse reactions may reduce concerns and help promote the vaccination of large population groups.

**Methods:**

All the reports of patients admitted to our Dermatology Primary Care, from March 2021 to June 2021, were retrospectively examined to collect descriptive data on skin reactions arising after COVID‐19 vaccination.

**Results:**

Out of 200 vaccinated patients admitted to the Dermatology Primary Care, 21 (10.5%) referred cutaneous reactions with onset after vaccination. Only one patient required hospitalization for generalized bullous erythema multiforme, which occurred 48 h after the second vaccine dose. The other patients' cutaneous reactions to vaccination were of mild/moderate degree. Three patients presented exacerbation of their cutaneous diseases.

**Conclusions:**

Cutaneous reactions observed in our sample were mostly mild or moderate. Awareness must be raised to recognize and treat eventual severe reactions. Future studies are needed to assess the incidence of cutaneous reactions following COVID‐19 vaccination.

## Graphical abstract

After COVID‐19 vaccination, pruritic rashes were frequently reported reactions at the Dermatology Primary care.

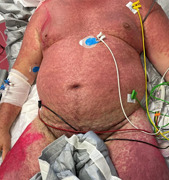

## 1 INTRODUCTION

Coronavirus disease 2019 (COVID‐19) vaccines are considered the most effective intervention to control the worldwide coronavirus epidemics we are nowadays confronted with.^1^

However, as for all types of drugs and vaccines, COVID‐19 vaccines can cause adverse reactions, mainly from vaccine‐induced immune responses.^1^, ^2^ Some of these may also involve the skin, often scaring unaware patients for the eye‐catching, unfamiliar presentation.^2^

A better understanding of such adverse reactions may reduce concerns and promote the vaccination of large population groups.

For this reason, to provide a report on COVID‐19 vaccine skin reactions, we collected all the patients' reports from our Dermatology Primary care to understand which cutaneous reactions were related to vaccination.

## 2 MATERIALS AND METHODS

From March 2021 to June 2021 all the patients admitted for any dermatological issue at the Dermatology Primary Care, after the first aid triage, were asked for recent COVID‐19 symptoms, exposures, and vaccination. All the reports were retrospectively examined to collect data on possible associations between cutaneous manifestations and COVID‐19 vaccination.

## 3 RESULTS

Of 200 patients (116 women, 84 men) admitted to the Dermatology Primary Care, who had been vaccinated against COVID‐19, 21 (10.5%) referred cutaneous reactions after COVID‐19 vaccination (Table 1).

**Table 1:** Features of patients, vaccines and cutaneous manifestations

Patient no	Age	Sex	Onset time from vaccination	Vaccination type	Dose no	Cutaneous manifestations	Other symptoms	Presumed COVID‐19 vaccine association
1	48	F	3 h	Oxford‐AstraZeneca	2nd	Small pruritic wheals on the upper limbs and chest	None reported	Correlated
2	27	F	4 h	BioNTech/Pfizer	2nd	Generalized maculopapular pruritic rash, with erythemtous patches on the knees	Headache	Correlated
3	29	F	12 h	Oxford‐AstraZeneca	1st	Facial swelling, flushing and erythema	Headache, nausea, asthenia	Correlated
4	76	M	24 h	Oxford‐AstraZeneca	1st	Maculopapular with micropapules and urticarial pruritic generalized rash	Headache, fever	Correlated
5	67	M	48 h	Moderna	2nd	Bullous pruritic erythema multiforme	Headache, fever, was hospitalized	Correlated
6	58	M	48 h	BioNTech/Pfizer	1st	Maculopapular pruritic generalized rash	None reported	Correlated
7	84	M	48 h	BioNTech/Pfizer	1st	Maculopapular pruritic generalized rash, starting from injection site	None reported	Correlated
8	35	F	72 h	Moderna	1st	Maculopapular pruritic generalized rash	Nausea, dyspnea, headache, vertigins	Correlated
9	27.	F	10 days	Oxford‐AstraZeneca	1st	Macular pruritic generalized rash (especially on neck, wrists, arms)	Headache	Correlated
10	43	F	10 days	BioNTech/Pfizer	1st	Urticarial pruritic generalized rash (especially on eyelids and arms)	None reported	Correlated
11	44	F	15 days	BioNTech/Pfizer	1st	Macular pruritic generalized rash with erythematous patches on the elbows	None reported	Correlated
12	59	F	15 days	BioNTech/Pfizer	2nd	Lichen planus plaques on both feet and ankles	None reported	Correlated
13	56	M	16 days	BioNTech/Pfizer	2nd	Psoriasis plaques on the trunk and limbs	None reported	Correlated
14	53	F	72 h	BioNTech/Pfizer	1st	Cutaneous sarcoidosis of the nose, eyelids, cheek	Dyspnea and pulmonary sarcoidosis	Correlated
15	58	F	20 days	BioNTech/Pfizer	2nd	Herpes Zoster, crural	Headache, asthenia	Correlated
16	82	F	20 days	BioNTech/Pfizer	2nd	Herpse Zoster, cervical	Generalized arthralgia and myalgia, asthenia, local dysesthesia	Correlated
17	37	F	30 days	Oxford‐AstraZeneca	2nd	Herpse Zoster, facial	Flu‐like symptoms	Correlated
18	31	M	30 days	BioNTech/Pfizer	2nd	Pityriasis Rosea, diffuse, only mildly pruritic	Headache, asthenia	Correlated
19	42	F	4 days	BioNTech/Pfizer	2nd	Ecchymosis on the third finger of the left foot	Headache, otalgia	Noncorrelated
20	54	F	24 h	BioNTech/Pfizer	2nd	Eruptive angiomas of trunk and arms	None reported	Noncorrelated
21	80	F	24 h	Oxford‐AstraZeneca	2nd	Giant seborrheic keratosis	None reported	Noncorrelated

Of these, 15 (71%) were females, with a mean age of 48 years, 6 (29%) were males, with a mean age of 62 years.

Female gender was not significantly more associated to reactions to the vaccine (4%, 15/116) than male gender (7%, 6/84) (*p* value >.05 calculated with Fisher exact test).

BioNTech/Pfizer was the most frequently reported vaccine used (62%, 13/21).

**Figure 1:**
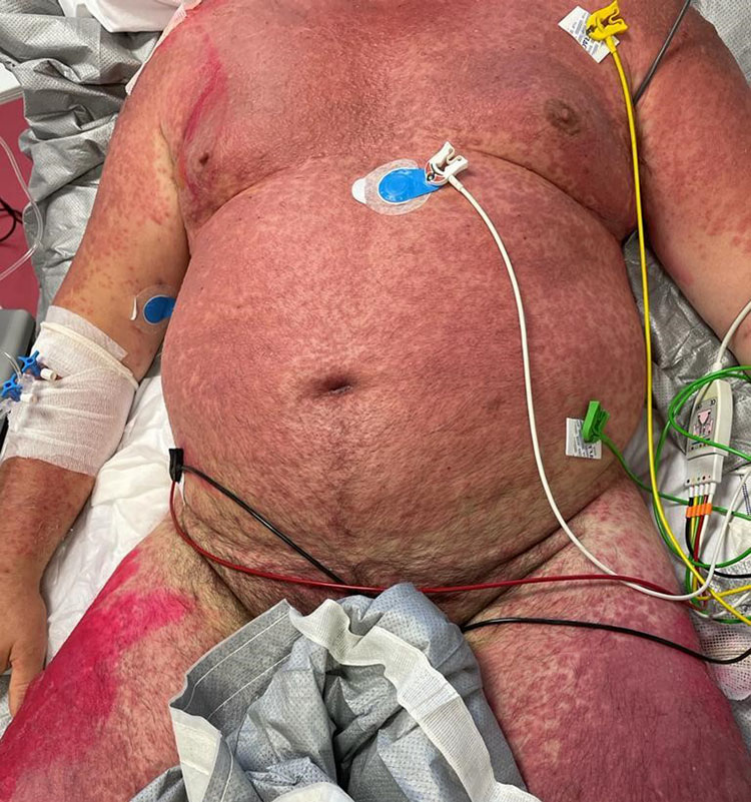
Severe diffuse bullous erythema multiforme with rapidly occurring and evolving, intensely red, round macules and papules, widely coalescing into large plaques and developing epidermal blistering

Reactions were more frequently reported after the second dose of vaccine (57%, 12/21) and occurred from 3 h to 30 days after vaccination. One patient out of 21, after the second dose of Moderna vaccine, developed a bullous erythema multiforme, which required hospitalization (Figure 1).

**Figure 2:**
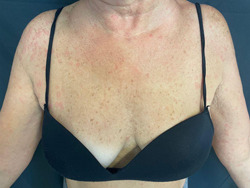
Urticarial reaction of the upper limbs and chest, characterized by small wheals with an erythematous rim and a pale center

Ten of 21 patients developed macular‐papular or urticarial reactions (Figure 2). In 4 patients out of 10, it appeared within 24 h of vaccine, in 6 patients after 24 h. Four out of 10 patients reported headaches associated with the rash. Two out of 10 patients reported nausea; only one had dizziness. The reaction developed after the first dose of vaccine in 6 of 10 patients.^3^

**Figure 3:**
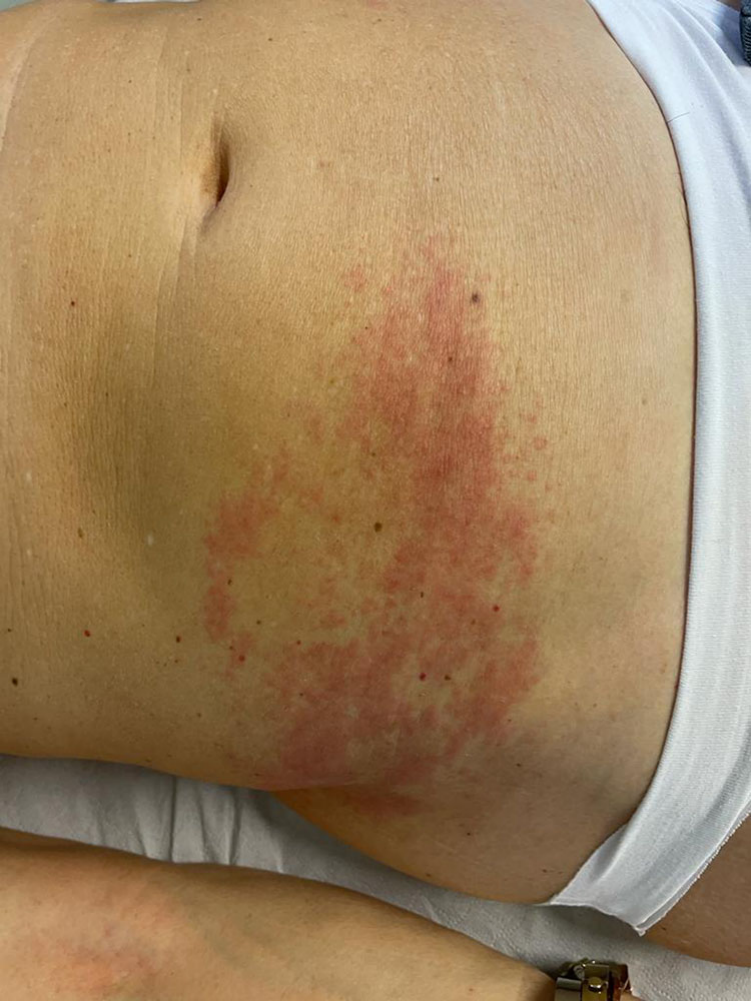
Mild localized unilateral Herpes Zoster, erythematous, blistering and painful rash caused by reactivation of varicella‐zoster virus, characterized by dermatomal distribution

Three out of 21 patients developed herpes zoster (HZ) 20 days after vaccination, associated with severe asthenia (Figure 3). None of the patients observed reported previous HZ. Only one patient out of 21 developed a diffuse form of pityriasis rosea of Gibert, only modestly itchy and associated with asthenia, after receiving the second dose of Pfizer vaccine.

**Figure 4:**
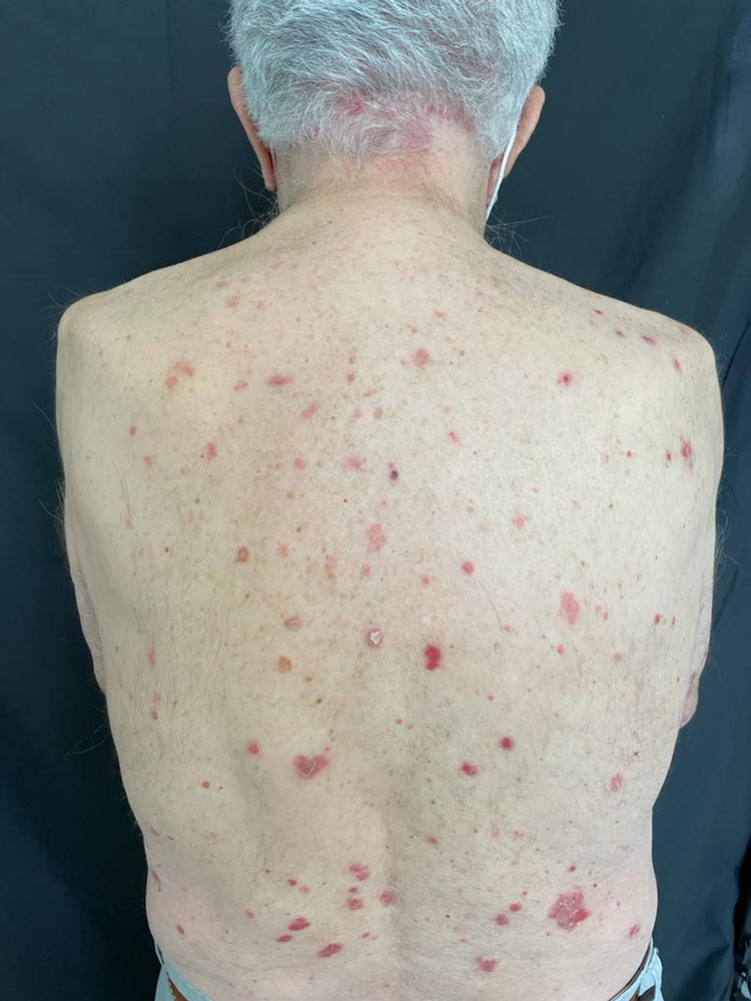
Recurrence of multiple psoriatic plaques, scaly, erythematous and slightly infiltrated, disseminated on the back

One patient reported the appearance of orange plaques on the nose, eyebrows and cheeks 72 h after administration of the first dose of Pfizer. A biopsy was performed and a diagnosis of sarcoidosis was made. One patient experienced a flare of lichen planus plaques^4^, 15 days after the second dose of Pfizer and one patient experienced recurrence of psoriatic plaques, 16 days after the second dose of Pfizer (Figure 4).

One patient out of 21 reported ecchymosis on the third toe of the left foot, 4 days after administration of Pfizer vaccine. One patient reported eruptive angiomas of the trunk and arms, which appeared within 24 h after the second dose of Pfizer vaccine. An 80‐year‐old patient claimed the appearance of a giant seborrheic keratosis within 24 h after the second dose of Pfizer vaccine.

## 4 DISCUSSION

Since COVID‐19 vaccination is spreading, there have been numerous reports of adverse reactions to the vaccine.^5^, ^6^, ^7^, ^8^, ^9^, ^10^, ^11^, ^12^, ^13^ The fear of the vaccine in the population is not justified, since many supposed reactions to the vaccine are in fact not due to it.^5^, ^6^ In our sample, only one of the 21 patients reporting skin reactions required hospitalization for generalized bullous erythema multiforme, which occurred 48 h after the second Moderna vaccine dose. Erythema multiforme is an immune‐mediated muco‐cutaneous inflammatory condition, which has been already reported in association with COVID‐19 and COVID‐19 vaccination, possibly caused by vaccine components eliciting an immune dysregulation, leading to a T‐lymphocyte auto‐immune response directed against keratinocytes, causing cell death and dermo‐epidermal junction detachment.^14^

Fortunately, none of the other patients developed life‐threatening reactions.

Ten out of 21 developed macular or urticarial rashes, which resolved with antihistamine or spontaneously, similarly to cases reported in the literature.^2^, ^3^, ^4^, ^5^, ^6^ Two patients presented generalized pruritic rash shortly after vaccination (≤4 h), possibly representing immediate hypersentivity type I reactions against vaccine excipients.^2^, ^3^, ^4^, ^5^, ^6^

Other rapid reactions reported were one pruritic generalized rash and a facial swelling and flushing, which occurred ≤24 h, but >4 h after vaccination, probably representing nonallergic (non‐immunoglobulin E mediated) but pseudo‐allergic reactions considering the time of occurrence.^5^, ^6^, ^7^, ^8^

The other 6 macular‐papular/urticarial reactions reported, occurred >48 h after vaccination, probably representing a delayed hypersensitivity (Coombs and Gell type IV) reactions.^8^, ^9^, ^10^

Though initially rarely observed during clinical approval studies, these hypersensitivity reactions seem now to be a well described phenomenon after COVID‐19 vaccination.^1^ Indeed, McMahon et al.^8^ reported in a recent study delayed large local reactions as the most frequent among cutaneous findings after messenger RNA (mRNA) COVID‐19 vaccinations (BioNTech/Pfizer and Moderna).

Of note, it must be considered that the rash may also represent an immune response to spike protein, as similar morbilliform eruptions that are negative for viral particles have been observed in patients with primary COVID‐19 infection.^15^

HZ virus reactivations were reported in 3 patients with a latency ≥20 days from vaccination, conceivably due to nonallergic vaccine immune dysregulation.^7^

All HZ cases reported in literature, following COVID‐19 vaccination, and our three cases, were moderate‐to‐mild cases and were successfully treated with antiviral therapy.^8^, ^16^, ^18^

Though the precise mechanisms involved in HZ development following COVID‐19 vaccinations are still unknown, the increasing reports of HZ reactivation after mRNA‐based and adenovirus‐based COVID‐19 vaccine call upon further studies.^8,^, ^17^, ^18^, ^19^, ^20^

Also, Pityriasis rosea (PR) (‐like) eruptions have been described after COVID‐19 vaccination. These may be secondary to a T‐cell‐mediated response, triggered by molecular mimicry from a viral epitope, or secondary to endogenous systemic reactivation of human herpes virus (HHV)‐6 and/or HHV‐7.^19^, ^20^, ^21^, ^22^, ^23^, ^24^ Of note, PR eruptions have been already described following other types of vaccinations, such as influenza vaccination with detection of HHV‐6 and HHV‐7 in skin biopsies.^19^, ^20^, ^21^, ^22^, ^23^

In our patient it occurred 30 days from the second dose of BioNTech/Pfizer and lasted approximately 35 days.

Interestingly, three patients presented exacerbation of their cutaneous diseases, including a flare of lichen planus, psoriasis and sarcoidosis, already present, and worsened, possibly induced by the vaccine.^25^

Last, three patients presented lesions that were discarded as not related to vaccination.

Indeed, seborrheic keratosis, eruptive angiomas, acral ecchymoses were interpreted as likely unrelated to the COVID‐19 vaccination and no literature reports were found to support possible causal associations.^25^

## 5 CONCLUSION

Despite the limitations of its descriptive and retrospective design, our study reports that cutaneous reactions to vaccination do not represent a contraindication to undergo COVID‐19 vaccination. These data are in line with the literature so far collected, although we did not record in our patients the most common reported reactions in the literature following COVID‐19 vaccines, i.e., local (early‐ or late‐onset) reactions and chilblain‐like lesions. This could possibly be the result of a selection bias, as the present study retrospectively assessed patients admitted at the Dermatology Primary Care.

Of note, cutaneous reactions considered worrisome by patients are mostly only mild or moderate, or not even associated with COVID‐19 vaccination, possibly suggesting patients are just apprehensive about anything they note after vaccination.

However, awareness must be raised to recognize and treat eventual severe reactions. As worldwide vaccination efforts are being adopted against COVID‐19, it is important for healthcare providers to recognize possible adverse events.

## FUNDING INFORMATION

Funding information is not available.

## CONFLICT OF INTERESTS

The authors declare that there are no conflict of interests.

## AUTHOR CONTRIBUTIONS

**Martina Burlando, Astrid Herzum, and Claudia Micalizzi:** performed the research. **Martina Burlando, Astrid Herzum, Claudia Micalizzi, Emanuele Cozzani, and Aurora Parodi:** designed the research study and revised the final version of the paper. **Martina Burlando, Astrid Herzum, and Emanuele Cozzani:** analyzed the data and wrote the paper. All authors have read and approved the final manuscript.
